# Towards Better Health at Work

**Published:** 1957-10

**Authors:** T. G. Faulkner Hudson

**Affiliations:** Lecturer in Industrial Hygiene, University of Bristol


### TOWARDS BETTER HEALTH AT WORK

BY

T. G. FAULKNER HUDSON, M.D., M.R.C.P.

*Lecturer in Industrial Hygiene, University of Bristol*

There is now in this country a working population of twenty four millions, the greatest number in our history. Every day the number of people away from employment for reasons attributed to ill-health averages one million, with an added sixty thousand due to injuries at work. Sickness absence accounts for 280 million working days lost yearly; injuries sustained when working accounts for another 18 million days, to which must be added $1\frac{1}{2}$ million days due to diseases directly due to the individual's occupation. Time lost owing to accidents sustained at work and to sickness exceed that due to industrial disputes seventy-five times. Industrial production must suffer when the producers are absent; living standards, including our standard of health care in a National Health Service, are threatened when national output declines. A healthy productive industrial community is an asset anywhere in the world: in this country is essential.

### NEED FOR INDUSTRIAL HEALTH SERVICE

The need of medical services at places of work is now hardly in dispute and their growth in recent years, while not spectacular, has been notable. The British Medical Association in 1941 recommended that at every factory, irrespective of size, there should be arrangements made for supervision of the workpeople. Four years later the Royal College of Physicians proposed the formation of a national industrial health service, applicable to every kind of employment, and made detailed recommendations for its organization. That such a service should be launched was also urged at about the same time by the Trades Union Congress. In 1949 the British Medical Association published its report on this matter in which considerable expansion of the then existing services was forecast. In the same year the Prime Minister appointed the Dale Committee, whose findings in 1951 included the recommendation that there should eventually be complete provision for occupational health covering not only industrial establishments of all kinds, both large and small, but also other occupations referred to by the Gowers Committee two years previously, after its enquiry into the health, welfare and safety in non-industrial employment. It was recognized at that time that this recommendation could not be made effective without first gathering more experience from surveys and experiments. At the end of 1954 the Minister of Labour and National Service stated his intention of developing health services in places covered by the Factories Acts and that the Committee especially set up to advise him would consider how best this should be done. Arising from this there are now two important field surveys being made; one is in the town of Halifax, chosen largely because of its many varied industries, and another amongst pottery workers whose occupation has long been associated with some risk to health.

It may therefore be concluded that while not all think the same about the ways by which medical services in industry should be extended, on the need of such extension there is unanimity of opinion. The political parties and the Trades Union Congress are not always in agreement; the same may perhaps be said of the British Medical Association and the Royal College of Physicians. When all of them think alike, it is indeed remarkable. Those who will be called upon to provide, use and pay for the proposed medical services should take notice.

### PRESENT FACILITIES

There is at present no comprehensive occupational medical service in this country, but there are many separate units. The Factory Department of the Ministry of

Labour and National Service has done important work for many years. The Factories Acts contain special requirements for promoting health at work, including a good working environment such as cleanliness, adequate sanitation, lighting, heating and ventilation, with protection of the workers against disease arising from work processes, and provision of first aid in case of sickness and injury. Under the control of the Chief Inspector of Factories there is the factory inspectorate with four specialist branches consisting of the medical, engineering, chemical and electrical inspectorates, all of whom are concerned with the prevention and detection of occupational disease and injury. The medical inspectors are helped by part time Appointed Factory Doctors who are usually general practitioners. In 1956 there were 16 whole time medical inspectors and 2,128 Appointed Factory Doctors, the duties of the latter being

(1) Medical examination for fitness for employment under the Factories Acts (including annual re-examination) of young persons under 18

(2) Statutory periodic medical examination of workers employed in certain unhealthy processes.

(3) Investigation and report on cases of industrial disease notifiable under the Factory Acts.

(4) Investigation and report on cases of death or injury caused by exposure to fumes or other noxious substances.

A small medical inspectorate covers the coalfields and quarries in Great Britain and is particularly concerned with all health measures applicable to those who work therein, as specified by the Mines and Quarries Act.

Government departments like the Treasury, the Ministries of Fuel and Power, and Supply have their own well organized medical services, as have also the nationalized industries such as the National Coal Board, Gas Boards, London Transport, British Railways, National Dock Labour Board and Atomic Energy Authority.

There are also many examples to be found of excellent medical care in private industrial companies, especially those of large size and with special health risks. In some cases, as in Slough, a group of small independent industries has formed a common health service, and these have proved very successful.

The number of factories which in 1948 had medical services other than statutory services is shown in Table 1.

Although nearly half the factories with more than 250 workers are seen to have some medical supervision in addition to statutory requirements, of the smaller factories employing the greater part of the factory population only about one per cent have any services beyond those required by law. Moreover, places of employment like shops, offices, hotels and catering establishments are not covered by the Factories Act, are not included in this table, and generally lack medical supervision. Therefore, although there has been development of medical work in industry in recent years, planning for the future must still be largely concerned with the needs of the many small working groups.

### THE INDUSTRIAL DOCTOR'S WORK

It is the task of the doctor in industry to advise management on all matters relating to health at work. He must be accessible to every member of the organization who wishes advice and must make a special study of the workpeople and of the conditions in which they work. A sound knowledge of chemistry, physics and engineering principles may be required. His duties are varied but he will always need good clinical judgement and the ability to gain the respect of both management and workpeople. A satisfactory relationship must be attained with family doctors, hospital staff and Public Health Officers. An efficient emergency casualty service for the treatment of those who become sick or injured at work must be organized. Continued medical care of conditions which can be treated to the patient's advantage while at work, should be carried out with the agreement of the patient's doctor. An analysis of the different kinds of work done should be made, assessing the demands

TABLE 1

| | Factories with | | | | | |
|---|---|---|---|---|---|---|
| | Less than 26 workers | 26–50 workers | 51–100 workers | 101–250 workers | Over 250 workers | Total |
| 1. Number of factories in Great Britain .. .. .. | 202,868 | 18,207 | 10,475 | 7,335 | 4,884 | 243,769 |
| 2. Number of factories with definite arrangements for medical services (other than statutory examinations of young persons) of one or more of the kinds indicated in 3 .. | 845 | 453 | 433 | 750 | 2,018 | 4,499 |
| 3. Kinds of Medical services in the factories in 2: | | | | | | |
| (*a*) General medical supervision over health of employees, including study of sickness records, working conditions, and to the firm on question of factory hygiene. .. | 230 | 137 | 223 | 418 | 1,517 | 2,525 |
| (*b*) Supervision of first aid or ambulance room services .. .. .. | 261 | 145 | 220 | 391 | 1,494 | 2,511 |
| (*c*) Periodical medical examinations (whether compulsory or not) of particular groups of workers, e.g. those in employment with special health risks. .. .. .. | 545 | 297 | 200 | 311 | 921 | 2,274 |
| (*d*) Periodical medical examinations of all workers | | | | | | |
| (i) at the factory .. | 27 | 13 | 20 | 50 | 119 | 229 |
| (ii) at the doctor's surgery .. .. .. | 4 | 1 | 4 | 3 | 6 | 18 |
| (*e*) Examination of new entrants and applicants for employment | | | | | | |
| (i) at the factory .. | 76 | 71 | 124 | 253 | 1,034 | 1,558 |
| (ii) at the doctor's surgery .. .. .. | 76 | 17 | 20 | 32 | 80 | 225 |
| (*f*) Examination of workers returning after illness | | | | | | |
| (i) at the factory .. | 46 | 46 | 83 | 184 | 800 | 1,159 |
| (ii) at the doctor's surgery .. .. .. | 68 | 7 | 11 | 21 | 34 | 141 |
| (*g*) Other kinds except statutory examination of young persons .. .. | 48 | 36 | 69 | 119 | 465 | 737 |

made by each on the worker; it then becomes possible to give an informed opinion regarding the work which can safely be undertaken, for example, by the young, the elderly and all who are temporarily or permanently incapacitated. In this way good rehabilitation can be provided at the place of work, and much unnecessary absence avoided. There are many harmful conditions met in industry which need recognition and control; they include dust, fume, large temperature change, excessive noise, poor illumination and ventilation and avoidable fatigue. The doctor may require help in his work from colleagues such as chemists, physicists and engineers, and as leader of the health team he must work in harmony with them. The doctor in industry must practise both preventive and curative medicine, but primarily it is his duty to anticipate trouble and prevent it, to detect occupational disease at its earliest stage, to investigate and eliminate all causes of disability and to attain a high standard of health in his community.

### WAYS TO ACHIEVE IMPROVEMENTS

For this work to progress, a new administrative framework will be necessary which the Government must initiate. A rapid introduction of medical services throughout industry at this time is not practicable; the extension of these services should be gradual and selective. There is a strong argument for acting quickly to reduce the burden of sickness which accounts for about three quarters of all absence from work, but a large and costly project making big demands on medical and nursing manpower, money and materials must be avoided. The standard of British practice in industrial health is high, perhaps the highest in the world, and this should be in mind when planning an advance. Much of medical practice today suffers from domination by party political interests; a further extension of this throughout industry, hampering management with faulty legislation supposed to improve health, would be a very serious matter and a costly failure. By legislation and much administrative change, of which a good deal has been heard, it might be possible before long to have a greatly increased number of treatment rooms in industry with doctors or nurses entering them from time to time in an attempt to exercise some kind of control. In such a scheme, which has its advocates there would no doubt be regional consultants appointed to supervise, a regional board to appoint them, and a Minister on top responsible to Parliament. No significant increase in health, however, would be likely to follow; in so far as the chief responsibility for health at work would be shifted onto the wrong shoulders, there might well be less. The promotion of occupational health is by no means wholly a medical matter. Employers, managers, trade unionists, physicists, chemists and engineers amongst others must be closely concerned in the matter. Employers are always likely to be better placed than doctors to improve health at work. It is they who select the people employed, who train (or do not train) them for allotted tasks, who decide the materials to be used and the method of use. It is they who control the working environment, upon which health so often depends. It is traditional in this country that the responsibility for health, safety and welfare of workpeople rests primarily with the employer, often advised by specialists, and always needing the interest and help of the workpeople themselves. This tradition is wholly good and should be strengthened. Although not always recognized, the interests of employers and employed concerning health at work are the same; when this is not yet accepted, it is the first task of the doctor in industry to see that it becomes so.

Professor I. G. Davies, Medical Officer of Health at Leeds, has drawn attention to the dangers of an artificial separation between medical care at work and at home. Any proposed industrial health plan should not create fresh divisions amongst doctors but should try to draw existing sections closer together. This has led to the suggestion of joining the statutory duties at places of work of the medical inspectors of mines and of factories, of the appointed Factory Doctors and of the Medical Officer of Health, thus making one preventive industrial health unit, and it is claimed that this would solve the chief problem of the small factory. A new medical inspectorate at central, regional and local levels would be created. The present medical inspectors of the Ministry of Labour and National Service would provide the regional level and one of their duties would be to make good contact with the administrative medical officer of the Regional Hospital Board and through him with the hospital services. At the local level it has been suggested that the Medical Officer of Health and his staff, assisted by the Appointed Factory Doctors should be responsible with the factory inspectorate for the enforcement of health standards and for medical examinations. A likely objection to a scheme of this kind is that it might lead to the handing over of much responsibility for industrial health to the Local Authorities, but possibly this could be avoided by seconding the

Medical Officer of Health, for his medical inspectorial functions, to the factory inspectorate without involving the Local Authority further. A stronger objection to this plan, however, is that for the promotion of health at work it relies too much on doctors who would be looked upon, whatever their title, as inspectors enforcing the law. There must always be some duties requiring an inspectorate but there is a point beyond which health cannot be imposed and we need an industrial climate in which those at work are encouraged to create for themselves conditions in which health can grow. Those industries, for instance, which have already provided good medical services should be encouraged to continue without any interference from outside bodies. An industrial health service should belong not to central or local government, but to those in industry who should be permitted some choice in selecting medical advice.

### THE PLAN OF PREVENTION

Remembering that the chief deficiency at places of work is not methods of treatment but prevention of disability, the outline of a proposed occupational health service can now be sketched. Of the several Ministries now concerned, the Ministry of Labour and National Service, with its Factory Department, has the greatest experience and its work in this field needs expansion. The Factory Department's medical inspectorate is very small in number and should be increased so that fuller attention can be given to the detection of occupational ill health which may now pass unrecognized and certainly unnotified. Help will be required from occupational hygiene laboratories which have been successfully provided in other countries such as the United States, Canada, Finland and Sweden, but which this country lacks. In these laboratories, of which 6-10 will be required, various specialists including doctors, chemists, engineers and physicists should work together, using their combined skills, with all necessary equipment, to assess and correct occupational hazards. They should be able to investigate problems submitted to them by the employer, industrial doctor, factory inspector or Medical Officer of Health. Air samples could be taken from the factory, dust counts made, radiation measured and surveys made of the lighting or ventilation. The laboratory staff would thus provide an important advisory service to industry on relevant matters of occupational health. There is a precedent in this country for such laboratories in the Public Health Laboratory Service, administered by the Medical Research Council, but the proposed occupational hygiene laboratories, which would not primarily be concerned with research, could probably be best placed under the control of the Factory Department of the Ministry of Labour and National Service. A few of the larger industries already have such laboratories for their own use, but a service of this kind for all occupational groups is urgently required. Industry itself might reasonably be expected to contribute towards the cost and some Universities would find such laboratories worthy of their support for their teaching value.

### POST-GRADUATE TRAINING FOR GENERAL PRACTITIONERS

The general practitioner, after some additional training for the special needs of industry, has a part to play much greater than at present. In Industry, as in the home, preventive and curative medicine can be practised to the advantage of both doctor and patient. It is already the policy of the government to encourage industrial managements to make their own arrangements for introducing medical advice and care at work, and three practical measures to further this object would be to grant some tax remission to those employers who do so, to make greater use of those sections of the Factories Acts whereby managements can be compelled to provide medical care when the conditions at work make this advisable, and to ensure that general practitioners and other doctors can without difficulty take short post-graduate courses in occupational health. The type of post-graduate instruction most likely to be useful is an intensive one lasting one or two weeks, these not being a substitute of course for full post-graduate training leading to a Diploma in Industrial Health. There has been demand for such courses but at present a general practitioner must not only pay the fee for the course but also provide for another doctor to do his practice work. Refresher courses are allowed to general practitioners under the National Health Service Act and similarly grants should be made available to them to cover the essential costs of attending an industrial health course. It has been suggested by the Occupational Health Committee of the British Medical Association that the course should be on the following lines:

*Objects*—To introduce doctors to medical work in industry so that they may use the time they devote to this work to the best advantage. In the one or two weeks available it is possible to give the newcomer a general idea of a doctor's work in industry. Techniques alone are not enough; an effort should be made to provide an essential background to the subject. In particular the opportunities for preventive work should be stressed.

*Content*—The following general subjects are suggested:

(1) Orientation: The aims of an occupational health service and its relation to the National Health Service and to other social services in the community.
(2) The Factory: Its functions, its organization, its management, and its personnel. The place of the doctor. The legal requirements for health, safety, and welfare. How to examine the working environment and assess working conditions, in co-operation with other experts. Other places of work will be dealt with similarly.
(3) Treatment at the place of work: Its nature and extent and the organizational and training problems involved. Accommodation and equipment.
(4) The nurse's work in industry.
(5) Medical examinations: their purpose and content.
(6) Records: what to keep and how to keep them; their purpose and value.
(7) Accidents in industry—industrial safety.
(8) The principles of the prevention of industrial disease illustrated in detail from local industries.
(9) Skin disease in industry.
(10) The care of special working groups—that is, young people, women, the elderly.
(11) The placement of the handicapped; the working of the Disabled Persons Employment Act.
(12) Clinical lectures on common diseases designed to emphasize the place of work in their management—that is, coronary disease, peptic ulcer, epilepsy, diabetes, etc.
(13) Sources of information—people, organizations, and literature.

*Method.* The following methods of teaching might be used:

(1) Lectures with questions and discussion.
(2) An occasional seminar if the composition of the class warrants it—that is, if it contains enough men with some experience of industrial medicine.
(3) Demonstrations—safety and rescue appliances.
(4) Visits: These should be specially selected and confined to places with a good occupational health department. The visits should be planned to illustrate specific subjects.
(5) Discussions (perhaps at places of work) with works manager, personnel manager, shop steward, if suitable people are available.

A course such as this could usefully be taken by doctors holding part-time occupational health appointments and by those about to take these up. Those who would take part in the teaching are experienced industrial medical officers, factory inspectors, some clinical teachers and others with special industrial experience, not necessarily with a medical qualification. At least six universities make provision for teaching of occupational health and it is there that these courses could best be arranged at first. Advanced and refresher courses should also be held, more often than at present, for those with greater experience of industry and those concerned with special health risks. Much occupational health could best be taught to medical students by clinical teachers, at the bedside and in the outpatient department, and those younger men and women, including registrars, who will later be responsible for undergraduate teaching should also be encouraged to attend these courses. At present too many students qualify with insufficient knowledge of medicine in its occupational aspects.

### USE OF MEDICAL AUXILIARIES

Apart from the work of doctors there is plenty of scope in industry for the employment of auxiliary nurses, and of men and women trained in giving of first aid. These last especially are required in greater numbers, whatever form their ordinary employment may take in the factory, being very useful in emergencies and in the reduction of accidents by their example in safe working methods. One of the duties of the doctor in industry is to ensure that these people are properly trained and encouraged to do this work efficiently.

### REGIONAL ADVISORY COMMITTEES

The Minister of Health has recently asked Regional Hospital Boards to appoint standing committees to advise on rehabilitation services locally but there is need of committees having wider scope and having within their terms of reference all matters concerning industrial health services, including rehabilitation. Committees with strong representation from doctors, industrial managements and the Unions already exist in some industrial centres in this country and have done useful work in advising local industries on health problems and on the introduction of medical services; so far progress has been made by voluntary effort only and government initiative now seems necessary, acting through the Ministry of Health and the Ministry of Labour and National Service.

### CONCERNING THE MEDICAL OFFICER OF HEALTH

Absence from work on account of illness of all kinds exceeds that directly due to occupations some 200 times. Little is known of the extent to which all this sickness is preventable and the proportion of it which arises in the home, workplace or elsewhere. It is anomalous that, although the chief duty of a Medical Officer of Health is to inform himself about all matters affecting, or likely to affect the public health, he is not at present concerned with industrial conditions apart from some relating to sanitation. He has very little opportunity of obtaining information which might enable him to improve the health of people at work; information derived from health visitors, district nurses, notification of infectious disease and the records showing applications of employed people for sickness benefit are together quite insufficient for this purpose. It has been more than once suggested in recent years that all employers of, say 25 people or more, in factories, catering establishments, offices or elsewhere, should notify the Medical Officer of Health monthly of the sickness incidence at the place of work. Information given would include the numbers employed, sex, the number absent for three consecutive days or more and (when known) the nature of the disability. The information would be incomplete but it should surely be of assistance to the Medical Officer of Health to know that absence from work in any establishment was high or low, the trend of it, and at least some of the reasons for it. He would then have facts regarding the sickness incidence in different occupations and should be able to follow up with further enquiries if this seemed advisable. Measures to prevent sickness have often followed the introduction of its notification and this proposal, if carried out, would probably lead to legislation insisting on satisfactory health standards at all places of work, similar to those now applying only to factories.

### SUMMARY

To summarize, it is believed that the need of an extension of medical care at work is accepted and that action to provide this should now be taken. Attention must especially be paid to the requirements of the smaller working groups and to those work places not covered by the Factories Acts. Priority should be given to the creation of a preventive, rather than a curative service and in this respect occupational hygiene laboratories with specialist staff and equipment are much needed. The imposition of a vast, expensive, bureaucratic service is unnecessary, likely to worsen and not better health, and must be resisted. The opportunity is presented of drawing together doctors and other specialists working in different fields so that they may apply their skills in preventing illness and promoting occupational health; this opportunity should be grasped. The law in some respects needs strengthening but health cannot be imposed on employers and workpeople, who must be encouraged to create for themselves conditions in which health can grow.

